# Omega-3 supplementation in patients with sepsis: a systematic review and meta-analysis of randomized trials

**DOI:** 10.1186/s13613-017-0282-5

**Published:** 2017-06-05

**Authors:** Clara Lu, Sunjay Sharma, Lauralyn McIntyre, Andrew Rhodes, Laura Evans, Saleh Almenawer, Lori Leduc, Derek C. Angus, Waleed Alhazzani

**Affiliations:** 1 Michael G. DeGroote School of Medicine, McMaster University, Hamilton, Canada; 2 Department of Surgery, Division of Neurosurgery, McMaster University, Hamilton, Canada; 3 Department of Medicine (Critical Care), The Ottawa Hospital Research Institute, University of Ottawa, Ottawa, Canada; 4 Department of Intensive Care Medicine, St George’s Hospital, Blackshaw Road, London, UK; 5 Department of Medicine, Division of Pulmonary Medicine and Critical Care, New York University, New York City, NY USA; 6 St. Joseph’s Healthcare Hamilton, 50 Charlton Avenue, L8N 4A6 Hamilton, ON Canada; 7 Department of Critical Care Medicine, University of Pittsburgh School of Medicine, Pittsburgh, PA USA; 8 Department of Health Research Methods, Evidence and Impact, McMaster University, Hamilton, Canada; 9 Department of Medicine, Division of Critical Care, McMaster University, Hamilton, Canada

**Keywords:** Omega-3, Fish oil, PUFA, EPA, DHA, Nutrition, Sepsis, Critical illness, ICU

## Abstract

**Background:**

Nutritional supplementation of omega-3 fatty acids has been proposed to modulate the balance of pro- and anti-inflammatory mediators in sepsis. If proved to improve clinical outcomes in critically ill patients with sepsis, this intervention would be easy to implement. However, the cumulative evidence from several randomized clinical trials (RCTs) remains unclear.

**Methods:**

We searched the Cochrane Library, MEDLINE, and EMBASE through December 2016 for RCTs on parenteral or enteral omega-3 supplementation in adult critically ill patients diagnosed with sepsis or septic shock. We analysed the included studies for mortality, intensive care unit (ICU) length of stay, and duration of mechanical ventilation, and used the Grading of Recommendations Assessment, Development and Evaluation approach to assess the quality of the evidence for each outcome.

**Results:**

A total of 17 RCTs enrolling 1239 patients met our inclusion criteria. Omega-3 supplementation compared to no supplementation or placebo had no significant effect on mortality [relative risk (RR) 0.85; 95% confidence interval (CI) 0.71, 1.03; *P* = 0.10; *I* ^2^ = 0%; moderate quality], but significantly reduced ICU length of stay [mean difference (MD) −3.79 days; 95% CI −5.49, −2.09; *P* < 0.0001, *I* ^2^ = 82%; very low quality] and duration of mechanical ventilation (MD −2.27 days; 95% CI −4.27, −0.27; *P* = 0.03, *I* ^2^ = 60%; very low quality). However, sensitivity analyses challenged the robustness of these results.

**Conclusion:**

Omega-3 nutritional supplementation may reduce ICU length of stay and duration of mechanical ventilation without significantly affecting mortality, but the very low quality of overall evidence is insufficient to justify the routine use of omega-3 fatty acids in the management of sepsis.

**Electronic supplementary material:**

The online version of this article (doi:10.1186/s13613-017-0282-5) contains supplementary material, which is available to authorized users.

## Background

Sepsis is a syndrome of life-threatening organ dysfunction caused by a dysregulated host response to infection. Mortality from sepsis is approximately 10% when the Sepsis-related Organ Failure Assessment (SOFA) score ≥2, and exceeds 40% in patients with septic shock [1]. Despite the advancement of best practice management by the Surviving Sepsis Campaign [2], the public health and disease burden of sepsis remains high [3–5]. As such, critical care research continues to search for ways to optimize clinical outcomes in this population, including through nutritional supplements [6].

Distinct changes in lipid metabolism have been noted in the critically ill, and the associations between nutritional intervention, lipid profile, and survival are of considerable interest [7]. Nutritional supplementation with omega-3 fatty acids has been proposed to modulate the immune response in critical illness by inhibiting pro-inflammatory (eicosanoid, NF-kB) and promoting anti-inflammatory (resolvin, protectin) mediators [8–11]. Clinical evidence for potential benefits of omega-3 fatty acids in acute respiratory distress syndrome (ARDS) [12, 13] and general critical illness [14, 15] has been tempered by studies showing equivocal effects [16–19] and even potential harm [20].

Several RCTs have also investigated omega-3 supplementation in sepsis over the past two decades; most recently, Hall et al. [21] suggested that a reduced ratio of arachidonic acid (AA) to eicosapentaenoic acid and docosahexaenoic acid [AA/(EPA + DHA)] after treatment with omega-3 fatty acids may be associated with improved survival in critically ill patients with sepsis. However, a comprehensive synthesis of these data has not been conducted, and the evidence for benefit remains unclear [2]. Therefore, we conducted a systematic review and meta-analysis of RCTs to evaluate the effect of omega-3 nutritional supplementation on clinical outcomes of adult critically ill patients with sepsis or septic shock.

## Methods

We did not publish or register a protocol for this systematic review.

### Eligibility criteria

Eligible studies met the following criteria: (1) randomized clinical trial (RCT) study design; (2) the population involved adult patients in the intensive care unit (ICU) with sepsis or septic shock; (3) the intervention group received either enteral or parenteral supplementation with omega-3 fatty acids; (4) the outcomes included any of the following: mortality (using the longest available follow-up time), ICU length of stay (LOS), and duration of mechanical ventilation (DMV).

### Search strategy

We searched MEDLINE, EMBASE, and the Cochrane Library from inception until December 2016. Our search strategies are given in Additional file 1: Tables S1–S3 and are limited to RCTs but not by language or publication date. We also screened the references from all included studies and relevant systematic reviews. Independently and in duplicate, two reviewers (CL and SS) screened titles and abstracts for eligibility, and conducted full-text reviews of selected studies. Disagreements over study selection were resolved by discussion and consensus. Studies fulfilling all of the eligibility criteria were included in the systematic review and meta-analysis.

### Data extraction

Two reviewers (CL and SS) independently extracted data of interest from included studies, with disagreements resolved by discussion and consensus. Mortality was the primary outcome, and ICU LOS and DMV were secondary outcomes. When data were missing or unclear, we contacted study authors for clarification.

### Risk of bias assessment

Using the Cochrane Collaboration tool [22], two reviewers (CL and SS) independently assessed each study for risk of bias in seven domains: random sequence generation, allocation concealment, blinding of patients and personnel, blinding of outcome assessment, incomplete outcome data, selective reporting, and other sources of bias. Disagreements were resolved by discussion and consensus, and adjudication by a third reviewer (WA) when necessary. For each study, the overall risk of bias was judged to be high if the risk of bias was high in any domain, unclear if the risk of bias was unclear in any domain (and not high in other domains), and low if the risk of bias was low across all domains.

### Statistical analysis

All analyses were performed using RevMan software (Review Manager, version 5.3. Copenhagen: The Nordic Cochrane Centre, The Cochrane Collaboration, 2014). We used inverse variance weighting and the DerSimonian and Laird [23] random-effects model to pool the weighted effect of estimates across studies. We reported relative risks (RRs) with 95% confidence interval (CI) for dichotomous outcomes and mean differences (MDs) with 95% CI for continuous outcomes. In studies where standard deviation (SD) for ICU LOS and DMV was not reported, we calculated SD from other measures of variability (standard error (SE), interquartile range (IQR), or 95% CI) following the methods suggested by the Cochrane Collaboration [24]. We combined low- and high-dose omega-3 intervention groups from one trial [25] into a single intervention group, using formulae described in the Cochrane Handbook [24] to calculate combined means and SDs for relevant outcomes. We assessed between-studies heterogeneity using Chi-square and *I* ^2^ statistics, with significant heterogeneity defined as *I* ^2^ > 50% or *P* < 0.10 [26].

We assessed publication bias for the mortality outcome by visual inspection of funnel plots. We investigated heterogeneity between studies by performing a post hoc subgroup analysis comparing parenteral with enteral administration of omega-3. We did not perform a subgroup analysis comparing risk of bias levels, as all studies had either a “high” or “unclear” overall risk of bias.

To explore the robustness of the results, we conducted the following post hoc sensitivity analyses: For all outcomes, we excluded trials that used per-protocol analysis, trials published in abstract form, trials that did not explicitly blind patients and healthcare personnel, trials that administered control formulae containing omega-3, and trials that did not administer the control group a placebo feed. For the mortality outcome, we excluded trials in which eligibility for inclusion was unclear (based on ICU admission), and further explored odds ratio (OR) as an alternative to RR analysis. For the ICU LOS outcome, we excluded trials that did not directly report SDs. Lastly for the DMV outcome, we excluded trials that did not directly report SDs and trials that did not stratify randomization by mechanical ventilation.

### Quality of evidence

For each outcome of interest, we used the Grading of Recommendations Assessment, Development and Evaluation (GRADE) approach [27] to rate the quality of evidence based on risk of bias, indirectness, inconsistency, imprecision, and other factors. Indirectness was evaluated by reviewing each trial’s study population, intervention, control, and outcomes. Inconsistency was evaluated using between-trial Chi-square and *I* ^2^ heterogeneity analyses. Imprecision was evaluated based on event rate, optimal sample size, and width of confidence intervals. The quality of evidence for each outcome was downgraded one level for “serious” limitations and two levels for “very serious” limitations.

## Results

### Search results

**Fig. 1:**
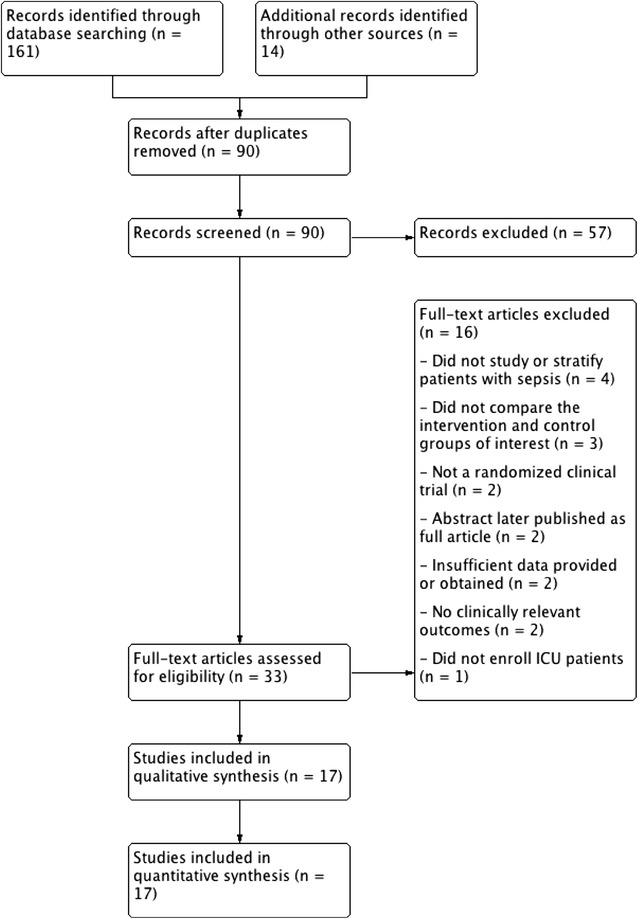
Preferred Reporting Items for Systematic Reviews and Meta-Analyses (PRISMA) flow chart: description of the study selection process. Seventeen trials (1 abstract and 16 fully published trials) were eligible and included in the qualitative and quantitative analyses. *ICU*, intensive care unit

Our search strategy identified a total of 175 citations, and 90 citations remained after removing duplicates. Screening of titles and abstracts led to the exclusion of 57 articles and the retrieval of 33 articles for full-text assessment, of which 16 were excluded for reasons outlined in Fig. 1. A total of 17 RCTs [25, 28–43] met our inclusion criteria, representing 1239 critically ill patients with sepsis.

### Study characteristics

Seventeen studies enrolled patients diagnosed with sepsis or subsets of sepsis (early sepsis [36], abdominal sepsis [32], severe sepsis [34, 35, 38], and septic shock [35, 38, 41]). Three studies [31, 35, 37] required both sepsis and mechanical ventilation for inclusion. One study [32] enrolled ICU and post-anaesthesia care unit (PACU) patients with abdominal sepsis; we abstracted data for the ICU subset only.

Ten studies [29, 32–34, 38–43] used the parenteral route to administer omega-3 supplements, while seven studies [25, 28, 30, 31, 35–37] used the enteral route. Brand-name formulae used included Omegaven [29, 32–34, 38, 41], Oxepa [31, 36, 37], Impact [28, 30], and Lipoplus [43]. Available data on the contents of all brand-name intervention and control formulae are given in Additional file 1: Tables S4–S5. Six studies calculated daily dose by weight [29, 31–33, 39, 40], five studies calculated daily dose by basal energy expenditure (BEE) and the Harris Benedict equation [28, 30, 35–37], and five studies administered a fixed daily dose [25, 34, 38, 41, 42]; in one study [43], the dosing method was not specified. The duration of supplementation ranged between 4 and 14 days.

While most studies administered the control group a placebo solution using standard enteral or parenteral formulae without omega-3, four studies [25, 29, 33, 41] assigned controls to standard sepsis care only; two of these explicitly defined standard care according to 2008 Surviving Sepsis Campaign guidelines [29, 33].

For outcomes, all studies assessed mortality, twelve assessed ICU LOS, and seven assessed DMV. Eleven studies evaluated 28-day mortality [25, 31, 33–36, 39–43], and the remainder defined mortality as 60-day [37], in-hospital [33], ICU [32], or left parameters undefined [28, 30, 38].

Six studies [28, 32, 34–36, 38] were appropriately blinded (patients, healthcare personnel, and research personnel), and seven studies [25, 29, 32, 33, 37, 39, 41] conducted a full intention-to-treat analysis of data. Six studies [28–31, 35, 36] were industry-funded. Finally, one study [32] was published solely as an abstract, but the authors provided missing data via personal communication. Table 1 presents further details of eligible studies.

**Table 1:** Characteristics of randomized clinical trials included

Study	Population	Intervention	Mortality definition	Risk of bias	Funding
Bower et al. [28] Multi-centre USA	ICU patients (*N* = 326); stratified by sepsis (*N* = 44 of 326) Age 18–80	Enteral administration Impact versus Osmolyte	NR	High	Pharmaceutical (Sandoz Nutrition)
Galban et al. [30] Multi-centre Spain	ICU patients with sepsis requiring EN (*N* = 181) Age > 14 (mean 55.8)	Enteral administration Impact versus Precitene Hiperproteico	NR	High	Pharmaceutical (Novartis Nutrition)
Grecu et al. [32] Single-centre Romania	ICU and PACU patients with abdominal sepsis requiring PN; 15 of 54 in ICU Age NR	Parenteral administration Omegaven + LCT versus LCT alone	ICU	Unclear	NR
Pontes-Arruda et al. [35] Single-centre Brazil	ICU patients with severe sepsis or septic shock requiring MV (*N* = 103) Age > 18 (mean 65.1)	Enteral administration EPA, GLA, antioxidants versus standard formulation	28-day	High	Pharmaceutical (Abbott Laboratories)
Guo et al. [42] Single-centre China	ICU patients with sepsis and APACHE II score >12 (*N* = 80) Age 18–70 (mean 40.0)	Parenteral administration Omega-3 PUFAs versus 20% fat emulsion	28-day	High	NR
Qu et al. [39] NR China	Patients with sepsis and APACHE II score 15–20 (*N* = 40) Age 18–65	Parenteral administration 10% omega-3 PUFAs versus standard TPN	28-day	Unclear	Academic. National Natural Science Foundation of China
Barbosa et al. [43] Single-centre Portugal	ICU patients with sepsis predicted to need parenteral nutrition (*N* = 25) Age range 32–80	Parenteral administration Lipoplus + NuTRIflex Special versus NuTRIflex Lipid Special	28-day and 5-day	High	No external funding
Wu et al. [40] Single-centre China	ICU patients with sepsis (*N* = 60) Mean age 63.42	Parenteral administration Omega-3 PUFAs versus 20% LCTs	28-day	High	NR
Zhao et al. [41] Single-centre China	ICU patients with sepsis or septic shock (*N* = 116) Mean age 53.3	Parenteral administration Omegaven versus standard care	28-day	Unclear	NR
Grau-Carmona et al. [31] Multi-centre Spain	ICU patients with sepsis and receiving MV (*N* = 132) Age ≥ 18 (mean 63)	Enteral administration Oxepa versus Ensure Plus	28-day	High	Pharmaceutical (Abbott Laboratories)
Khor et al. [34] Single-centre Taiwan	ICU patients with severe sepsis (*N* = 28) Age ≥ 18 (mean 69.3)	Parenteral administration Omegaven versus normal saline	28-day	Unclear	NR
Pontes-Arruda et al. [36] Multi-centre Brazil	ICU patients with early sepsis requiring EN (*N* = 106) Age > 18 (mean 71)	Enteral administration Oxepa versus Ensure Plus HN	28-day	High	Pharmaceutical (Abbott Laboratories)
Hosny et al. [25] Single-centre Egypt	ICU patients with early sepsis (*N* = 75) Age ≥ 18 (mean 52.1)	Enteral administration (oral or NG) High-dose omega-3 + antioxidants versus low-dose omega-3 + antioxidants versus standard care	28-day	High	NR
Burkhart et al. [29] Single-centre Switzerland	ICU patients with sepsis (*N* = 50) Age > 18 (mean 69)	Parenteral administration Omegaven versus standard care	Median follow-up 109 days	High	Pharmaceutical (Fresenius Kabi) and academic
Gultekin et al. [38] Single-centre Turkey	General Surgery ICU patients with severe sepsis or septic shock requiring TPN (*N* = 32) Age ≥ 18 (mean 62.9)	Parenteral administration Omegaven + ClinOleic—Baxter olive oil emulsion versus olive oil emulsion	NR	High	NR
Hall et al. [33] Single-centre United Kingdom	ICU patients with sepsis (*N* = 60) Adults (mean age 64.2)	Parenteral administration Omegaven versus standard care	28-day and inpatient	Unclear	Omegaven supplied by Fresenius Kabi; no other financial support
Shirai et al. [37] Single-centre Japan	ICU patients with sepsis-induced ARDS receiving MV (*N* = 46) Age > 18 (mean 72.5)	Enteral administration Oxepa versus Ensure Liquid	60-day	High	NR

*APACHE II* Acute Physiology and Chronic Health Evaluation II, *ARDS* acute respiratory distress syndrome, *EN* enteral nutrition, *EPA* eicosapentaenoic acid, *GLA* gamma linolenic acid, *ICU* intensive care unit, *LCT* long-chain triglycerides, *MV* mechanical ventilation, *NG* nasogastric, *NR* non-reported, *PN* parenteral nutrition, *PUFAs* polyunsaturated fatty acids, *TPN* total parenteral nutrition

### Risk of bias

**Fig. 2:**
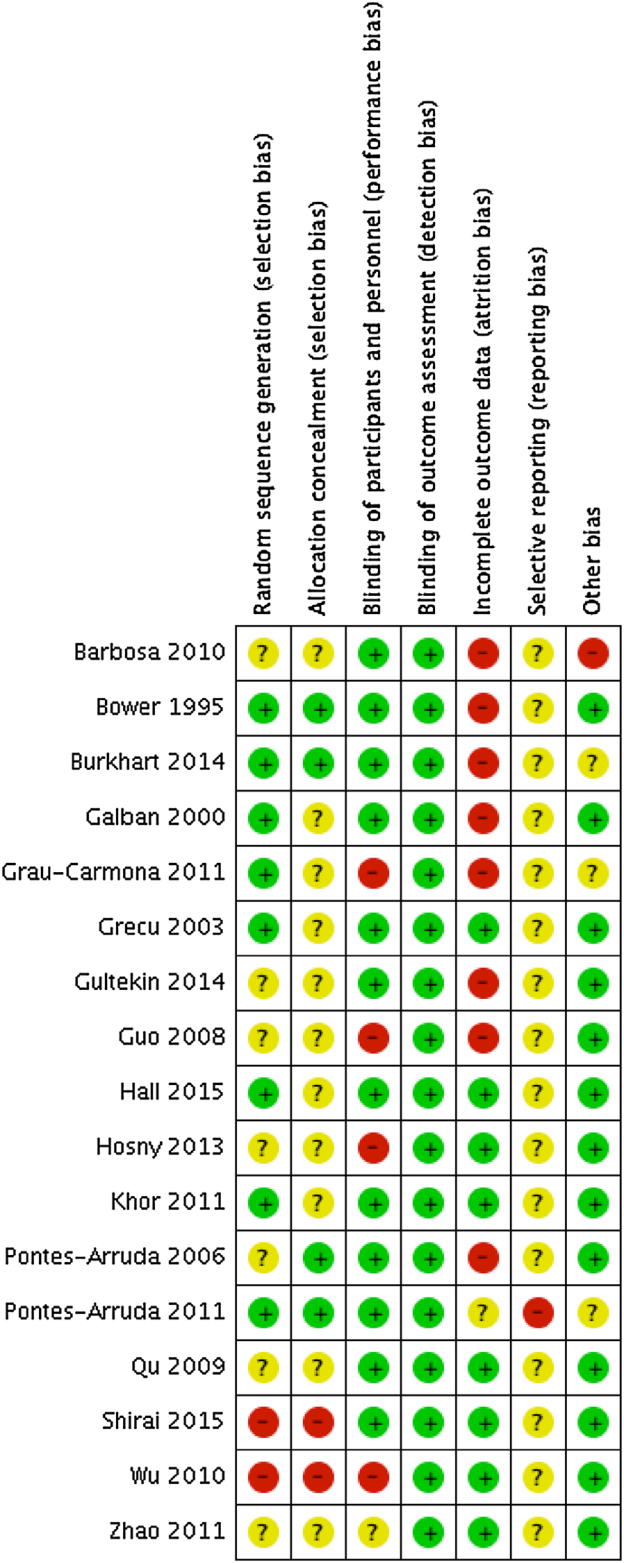
Risk of bias assessment of the included trials using the Cochrane Collaboration tool. Individual risk of bias assessments across seven domains: random sequence generation, allocation concealment, blinding of participants and personnel, blinding of outcome assessment, incomplete outcome data, selective reporting, and other bias. Risk of bias levels: low (*green*), unclear (*yellow*), high (*red*)

Using the Cochrane Collaboration tool for risk of bias [22], twelve studies were judged to be at high risk of bias, many of these due to attrition and performance bias. Risk of bias was unclear for the remaining five studies (Fig. 2).

### Quality of evidence

Table 2 presents our GRADE [27] assessment of the quality of evidence by outcome. Evidence quality was assessed as moderate for mortality and very low for both ICU LOS and DMV outcomes.

**Table 2:** Grading of Recommendations Assessment, Development and Evaluation (GRADE) evidence profile

Quality assessment						No. of patients		Effect		Quality
No. of studies	Risk of bias	Inconsistency	Indirectness	Imprecision	Other considerations	Omega-3 supplementation	Control	Relative (95% CI)	Absolute (95% CI)	
Mortality										
17	Not serious	Not serious	Serious^a^	Not serious^b^	None	149/629 (23.7%)	161/610 (26.4%)	RR 0.85 (0.71 to 1.03)	40 fewer per 1000 (from 8 more to 77 fewer)	Moderate
ICU length of stay										
12	Serious^c^	Serious^d^	Serious^a^	Not serious	None	469	456	–	MD 3.79 days fewer (5.49 fewer to 2.09 fewer)	Very low
Duration of mechanical ventilation										
7	Serious^c^	Serious^e^	Serious^a^	Not serious	None	254	241	–	MD 2.27 days fewer (4.27 fewer to 0.27 fewer)	Very low

*CI* confidence interval, *RR* relative risk, *MD* mean difference

^a^We rated down the quality of evidence by one level for multiple sources of indirectness. Population: mechanical ventilation and sepsis severity varied as inclusion criteria across studies. Intervention: content of enteral/parenteral formulations differed across studies (10 used fish oil alone while 7 used formulae with additional supplements such as mRNA, arginine, and selenium). Outcome: different mortality definitions (28-day, 60-day, in-hospital, ICU)

^b^We did not rate down the quality of evidence for imprecision. The CI included both significant benefit and small harm, but the number of events was not small

^c^We rated down the quality of evidence by one level for risk of bias. Several studies showed high risk of attrition bias and performance bias

^d^We rated down the quality of evidence by one level for significant unexplained heterogeneity (*P* < 0.00001, *I* ^2^ = 82%)

^e^We rated down the quality of evidence by one level for significant unexplained heterogeneity (*P* = 0.02, *I* ^2^ = 60%)

### Main outcomes

**Fig. 3:**
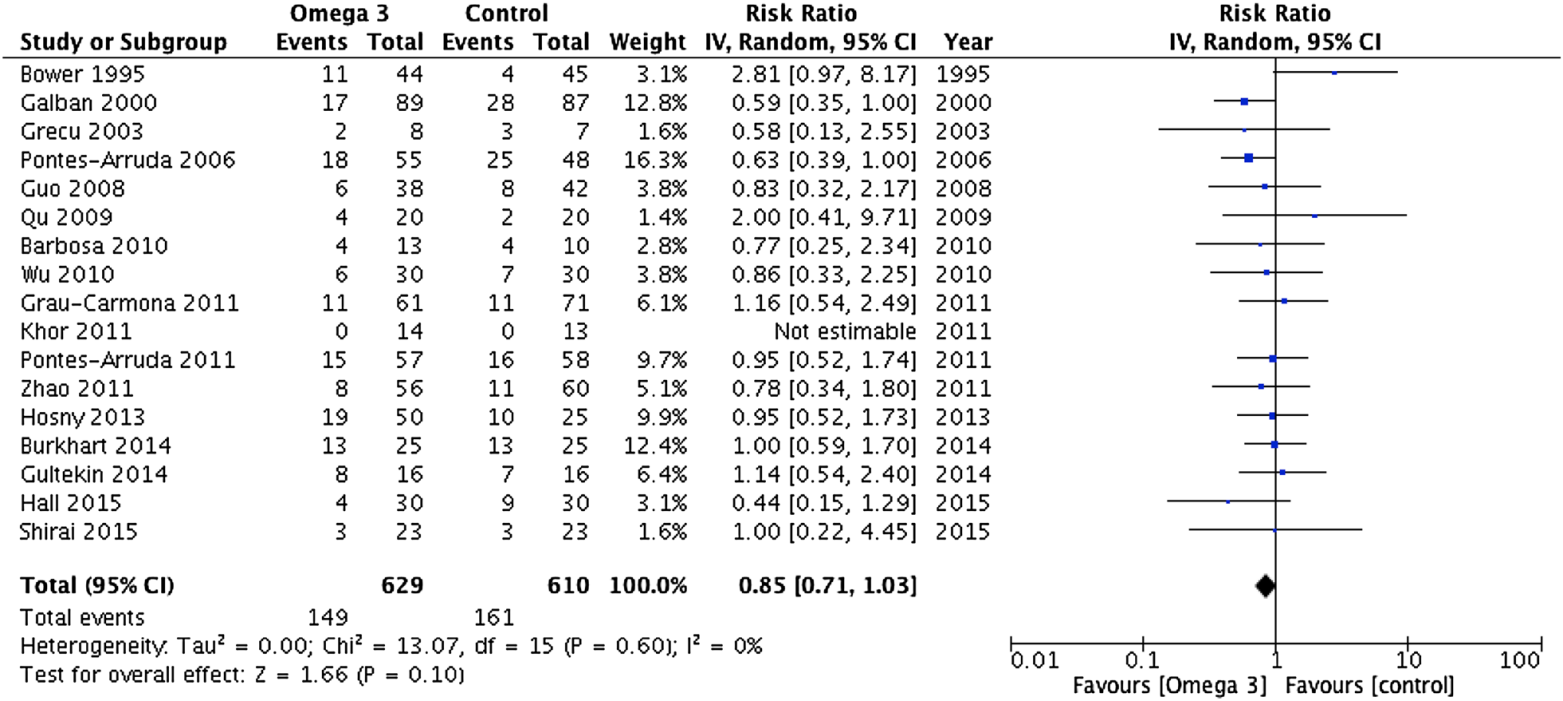
Mortality outcome. Data from 17 trials (*n* = 1239 patients) were included and analysed using the random-effects model. Omega-3 supplementation was associated with a non-significant reduction in mortality. *ICU* intensive care unit, *IV* inverse variance, *RCT* randomized clinical trial

**Fig. 4:**
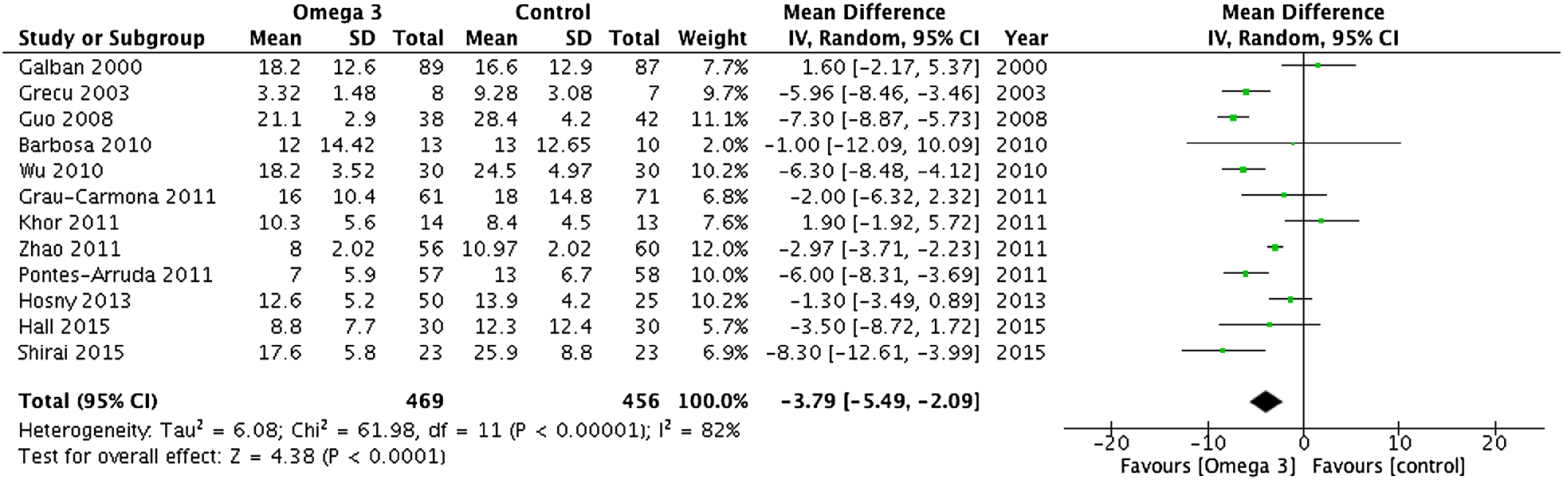
ICU length of stay outcome. Data from 12 trials (*n* = 925 patients) were included and analysed using the random-effects model. Omega-3 supplementation was associated with a significantly lower length of stay in ICU. *IV* inverse variance

**Fig. 5:**
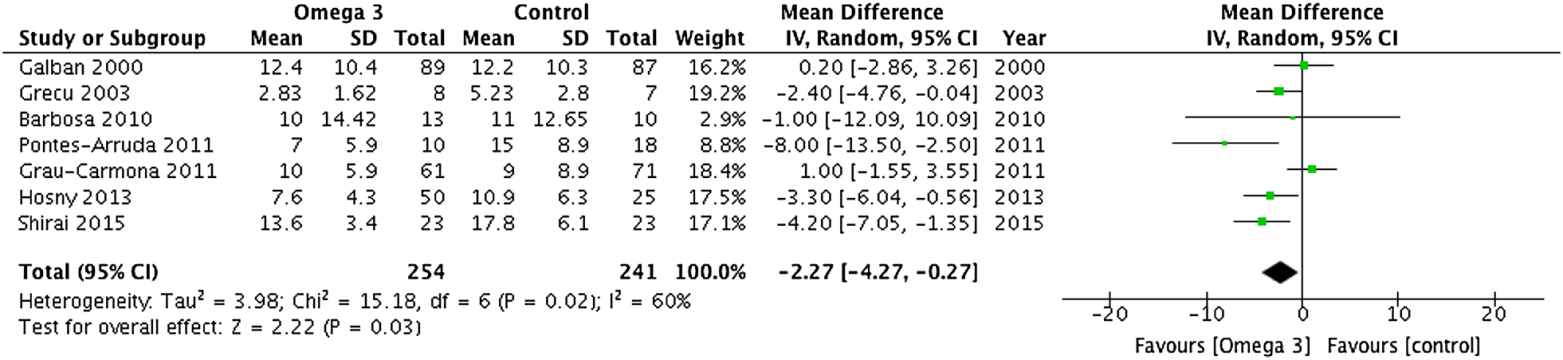
Duration of mechanical ventilation outcome. Data from 7 trials (*n* = 495 patients) were included and analysed using the random-effects model. Omega-3 supplementation was associated with a significantly shorter duration of mechanical ventilation. *IV* inverse variance

Mortality was reported by 17 trials enrolling 1239 patients (Fig. 3). Omega-3 was not associated with a significant reduction in mortality (RR 0.85; 95% CI 0.71, 1.03; *P* = 0.10; *I* ^2^ = 0%; moderate quality). ICU length of stay was reported in 12 trials enrolling 925 patients (Fig. 4). There was a significant reduction in ICU LOS (MD −3.79 days; 95% CI −5.49, −2.09; *P* < 0.0001, *I* ^2^ = 82%; very low quality) for patients supplemented with omega-3. Duration of mechanical ventilation was reported in seven trials enrolling 495 patients (Fig. 5). There was a significant reduction in DMV (MD −2.27 days; 95% CI −4.27, −0.27; *P* = 0.03, *I* ^2^ = 60%; very low quality) in the group of patients supplemented with omega-3.

### Subgroup and sensitivity analyses

Ten studies used parenteral administration and seven studies used enteral administration of omega-3. We performed a post hoc subgroup analysis comparing these subgroups for the outcome of mortality (Additional file 1: Figure S1) and found no significant differences in treatment effect (*P* = 0.97, *I* ^2^ = 0%) between trials that used parenteral (RR 0.89; 95% CI 0.66, 1.19; *P* = 0.42; *I* ^2^ = 0%) compared to enteral (RR 0.88; 95% CI 0.64, 1.21; *P* = 0.43; *I* ^2^ = 35%) routes. However, the analysis is confounded by another major difference between subgroups: Most enteral formulations administered omega-3 in combination with other supplements (including arginine, selenium, and mRNA), while all parenteral formulations administered omega-3 as the sole supplement. Since the distinct influences of these two characteristics (additional supplementation and route of administration) on the treatment effect cannot be distinguished, no definitive conclusions can be drawn from this analysis.

Sensitivity analyses excluding trials that used per-protocol analysis [28, 30, 31, 34–36, 38, 40, 42, 43] and trials that used control formulae containing omega-3 [31, 36] produced congruent results for all three outcomes. For the mortality outcome, exclusion of trials in which ICU admission was unclear [39], trials published in abstract form [32], trials without explicit blinding of patients and healthcare personnel [25, 29–31, 33, 37, 39–43], and trials that did not administer the control group a placebo feed [25, 29, 33, 41] yielded similar non-significant results.

However, for the ICU LOS outcome, exclusion of trials that did not explicitly blind patients and healthcare personnel to the intervention [25, 30, 31, 33, 37, 40–43] rendered the significant reduction in ICU LOS non-significant (MD −3.63; 95% CI −7.85, 0.60; *P* = 0.09, *I* ^2^ = 85%). Similarly, the significant reduction in DMV was countered by the exclusion of trials that did not explicitly blind patients and healthcare personnel [25, 30, 31, 37, 43] (MD −4.63; 95% CI −10.00, 0.75; *P* = 0.09, *I* ^2^ = 70%), trials that did not stratify mechanically ventilated patients during randomization [25, 30, 36] (MD −1.78 days; 95% CI −4.39, 0.83; *P* = 0.18, *I* ^2^ = 61%), trials that did not administer the control group a placebo feed [25] (MD −2.10 days; 95% CI −4.48, 0.29; *P* = 0.08, *I* ^2^ = 65%), and trials published in abstract form [32] (MD −2.32 days; 95% CI −4.86, 0.22; *P* = 0.07, *I* ^2^ = 67%). Details of these analyses are given in Additional file 1: Tables S6–S8.

### Publication bias

Visual inspection of funnel plots for the mortality and ICU LOS outcomes (Additional file 1: Figures S2–S3) did not reveal small-study effects suggestive of publication bias.

## Discussion

Our meta-analysis of 17 RCTs (1239 patients) suggests, based on moderate-quality evidence, that omega-3 supplementation does not significantly reduce mortality in septic critically ill patients, with the absolute effect ranging from 77 fewer to 8 more deaths per 1000 patients. Very low-quality evidence also suggests that omega-3 may reduce length of ICU stay and duration of mechanical ventilation, but these results are challenged by multiple sensitivity analyses.

A recent meta-analysis of eleven RCTs (808 patients) [44] also explored omega-3 in the critically ill with sepsis and similarly found a non-significant reduction in mortality (RR 0.84; 95% CI 0.67 to 1.05; *P* = 0.12) and a significant reduction in DMV (MD −3.82 days; 95% CI −4.61 to −3.04; *P* < 0.001). However, the authors did not find a significant reduction in ICU LOS (MD −2.70 days; 95% CI −6.40 to 1.00; *P* = 0.15). Key differences in their analysis include the search of a single database (PubMed), the use of the Jadad score to assess risk of bias [45], the absence of six studies included in our analysis [25, 28, 39–42], and the inclusion of one study excluded from our analysis for enrolling patients without sepsis [46].

Another meta-analysis of twelve RCTs (721 patients) evaluated the effects of parenteral omega-3 in sepsis [47]. Their analysis revealed a significant reduction in 28-day mortality (RR 0.77, 95% CI 0.59 to 0.99, *P* = 0.04) and ICU LOS (MD −3.10 days; 95% CI −5.98 to −0.21; *P* = 0.04) and a non-significant effect on DMV (MD 1.33 days; 95% CI −5.09 to 7.75; *P* = 0.69). Here the inconsistencies with our meta-analysis may be explained by the exclusion of trials using enteral omega-3, the inclusion of one study excluded from our analysis for enrolling patients without sepsis [46], and the absence of more recently published RCTs [29, 33, 38].

Beyond the potential reductions in DMV and ICU LOS suggested by the present meta-analysis, the risks and costs of omega-3 supplementation must also be addressed. Although the cost of omega-3 supplementation varies by dose, frequency, route of administration, and choice of formula, the leading parenteral formula used in this meta-analysis (Omegaven) has been reported to cost up to 3 times more than another lipid emulsion in conventional use [48]. According to its manufacturer, Omegaven in North America is currently obtained only by applying to special access programs.

Safety is another key consideration. While most RCTs studying omega-3 supplementation in critical illness have reported minimal adverse effects, others have identified important risks that include significantly longer hospital and ICU lengths of stay [30, 34], increased duration of mechanical ventilation [47], fewer ventilator-free and ICU-free days [20], elevated triglyceride levels [29, 37], and a higher incidence of diarrhoea [20, 25]. Most concerning are reported trends towards increased mortality [20, 28, 31, 39, 49]. Even in meta-analyses that demonstrate a non-significant reduction in mortality [18, 44, 50, 51], as this one does, the upper limit of the CI cannot exclude the potential for increased mortality with omega-3 supplementation. Detailed data on non-surviving patients would be necessary to explore characteristics associated with increased mortality risk with omega-3 supplementation.

Limitations that call for cautious interpretation of these findings exist at both the study and review level. Dosing and route of administration varied across trials; whether these characteristics modify the treatment effect has not been sufficiently studied for omega-3, and our subgroup analysis comparing enteral and parenteral routes was inconclusive. Recognizing that several trials administered omega-3 in combination with other supplements, we downgraded the quality of evidence across outcomes for indirectness of intervention. For ICU LOS and DMV, we further downgraded the quality of evidence for significant heterogeneity and high overall risk of bias.

Strengths of this meta-analysis include its comprehensive database search, literature assessments conducted independently and in duplicate, the expertise of a registered dietician, the use of the Cochrane Collaboration Tool [22] to assess risk of bias, and careful adherence to the GRADE approach [27] and PRISMA guidelines [52]. It addresses a specific question and includes recent eligible trials. To date, this is the largest meta-analysis conducted on the effect of omega-3 supplementation in critically ill patients with sepsis.

## Conclusions

Our meta-analysis should prompt caution against the routine use of omega-3 fatty acid supplementation for critically ill patients with sepsis. While very low-quality evidence suggests that omega-3 may reduce the number of days patients spend on mechanical ventilation and in the ICU, these effects are overturned by multiple sensitivity analyses. Moderate-quality evidence also demonstrates a non-significant trend towards reduced mortality, yet the upper limit of confidence reveals potential for harm. Here, even the slightest possibility of increased mortality (moderate-quality evidence) demonstrated in the present and previous meta-analyses still outweighs the potential benefits of reduced ICU LOS and DMV (very low-quality evidence).

With current evidence limited in quality and quantity, the profile of risk and benefit does not favour treatment of sepsis with omega-3. Justification for omega-3 in sepsis will require large-scale, high-quality RCTs that strengthen the evidence for clinical benefit enough to outweigh the risks and costs of this intervention [53]. Until then, the routine use of omega-3 fatty acid supplementation in patients with sepsis should be avoided.

## Additional file

**Additional file 1: Table S1.** Search Strategy—MEDLINE.** Table S2.** Search Strategy—EMBASE.** Table S3.** Search Strategy—Cochrane Library.** Table S4.** Contents of Brand-name Parenteral Formulations.** Table S5.** Contents of Brand-name Enteral Formulations.** Figure S1.** Subgroup Analysis for Mortality Outcome.** Table S6.** Sensitivity Analyses for Mortality Outcome.** Table S7.** Sensitivity Analyses for ICU Length of Stay Outcome.** Table S8.** Sensitivity Analyses for Duration of Mechanical Ventilation Outcome.** Figure S2.** Funnel Plot for Mortality Outcome.** Figure S3.** Funnel Plot for ICU Length of Stay Outcome.** Table S9.** PRISMA Checklist.

## Abbreviations

AA: arachidonic acid
APACHE II: Acute Physiology and Chronic Health Evaluation II
ARDS: acute respiratory distress syndrome
BEE: basal energy expenditure
CI: confidence interval
DHA: docosahexaenoic acid
DMV: duration of mechanical ventilation
EN: enteral nutrition
EPA: eicosapentaeonic acid
FE: fixed effects
GLA: gamma linolenic acid
GRADE: Grading of Recommendations Assessment, Development and Evaluation
ICU: intensive care unit
ITT: intention to treat
LCT: long-chain triglycerides
LOS: length of stay
MD: mean difference
MV: mechanical ventilation
NG: nasogastric
NR: non-reported
OR: odds ratio
PACU: post-anaesthesia care unit
PN: parenteral nutrition
PRISMA: Preferred Reporting Items for Systematic Reviews and Meta-Analyses
PUFA: polyunsaturated fatty acid
RCT: randomized clinical trial
RE: random effects
RR: relative risk
SOFA: Sepsis-related Organ Failure Assessment
TPN: total parenteral nutrition
