# Supplementary material for: Omega-3 supplementation in patients with sepsis: a systematic review and meta-analysis of randomized trials
Source: Ann Intensive Care. 2017 Jun 5;7:58. doi: 10.1186/s13613-017-0282-5 (PMC5459780; doi:10.1186/s13613-017-0282-5)
Supplement: Supplementary file 1 — Additional file 1: Table S1. Search Strategy—MEDLINE. Table S2. Search Strategy—EMBASE. Table S3. Search Strategy—Cochrane Library. Table S4. Contents of Brand-name Parenteral Formulations. Table S5. Contents of Brand-name Enteral Formulations. Figure S1. Subgroup Analysis for Mortality Outcome. Table S6. Sensitivity Analyses for Mortality Outcome. Table S7. Sensitivity Analyses for ICU Length of Stay Outcome. Table S8. Sensitivity Analyses for Duration of Mechanical Ventilation Outcome. Figure S2. Funnel Plot for Mortality Outcome. Figure S3. Funnel Plot for ICU Length of Stay Outcome. Table S9. PRISMA Checklist. [file 13613_2017_282_MOESM1_ESM.docx]

**Omega-3 Supplementation in Patients with Sepsis: A Systematic Review and Meta-analysis of Randomized Trials**

Clara Lu^1^, Sunjay Sharma^2^ , Lauralyn McIntyre^3^, Andrew Rhodes^4^, Laura Evans^5^, Saleh Almenawer^2^, Lori Leduc^6^, Derek C. Angus^7^, Waleed Alhazzani^8,9^

1. Michael G. DeGroote School of Medicine, McMaster University, Hamilton, Canada
2. Department of Surgery, Division of Neurosurgery, McMaster University, Hamilton, Canada
3. Department of Medicine (Critical Care), The Ottawa Hospital Research Institute, University of Ottawa, Ottawa, Canada
4. Department of Intensive Care Medicine, St George's Hospital, Blackshaw Road, London, UK
5. Department of Medicine, Division of Pulmonary Medicine and Critical Care, New York University, New York City, USA
6. St. Joseph’s Healthcare, Hamilton, Canada
7. Department of Critical Care Medicine, University of Pittsburgh School of Medicine, Pittsburgh, USA
8. Department of Clinical Epidemiology and Biostatistics, McMaster University, Hamilton, Canada
9. Department of Medicine, McMaster University, Hamilton, Canada

**Correspondence**

Waleed Alhazzani MD, MSc, FRCPC

McMaster University, Department of Medicine, Division of Critical Care

St Joseph’s Healthcare Hamilton

50 Charlton Avenue, Postal Code L8N 4A6, Hamilton, Ontario, Canada

Tel: +1905-522-1155 ext 32800

Fax: +1905-521-6068

Email: [alhazzaw@mcmaster.ca](mailto:alhazzaw@mcmaster.ca)

**Table of Contents**

| page  3  5  7  8  9  11  12  13  14  15  16  17 |
| --- |

**Table 1.** Search Strategy – MEDLINE

**Table 2.** Search Strategy – EMBASE

**Table 3.** Search Strategy – Cochrane Library

**Table 4.** Contents of Brand-name Parenteral Formulations

**Table 5**. Contents of Brand-name Enteral Formulations

**Figure 1.** Subgroup Analysis for Mortality Outcome

**Table 6.** Sensitivity Analyses for Mortality Outcome

**Table 7.** Sensitivity Analyses for ICU Length of Stay Outcome

**Table 8.** Sensitivity Analyses for Duration of Mechanical Ventilation Outcome

**Figure 2.** Funnel Plot for Mortality Outcome

**Figure 3.** Funnel Plot for ICU Length of Stay Outcome

**Table 9.** PRISMA Checklist

**Table 1.** Search Strategy for MEDLINE

1 sepsis.mp. or exp Sepsis/ or exp Shock, Septic/ (167568)

2 septic*.mp. (81994)

3 1 or 2 (200978)

4 enteral nutrition.mp. or exp Enteral Nutrition/ (21189)

5 enteral feed*.mp. (5807)

6 enteric feed*.mp. (103)

7 feeding tube*.mp. (3431)

8 tube feed*.mp. (3734)

9 4 or 5 or 6 or 7 or 8 (26181)

10 parenteral nutrition.mp. or exp Parenteral Nutrition/ (30522)

11 parenteral feed*.mp. (1704)

12 intravenous feed*.mp. (492)

13 iv feed*.mp. (58)

14 total parenteral nutrition.mp. or exp Parenteral Nutrition, Total/ (14089)

15 total nutrient admixture*.mp. (99)

16 10 or 11 or 12 or 13 or 14 or 15 (30984)

17 9 or 16 (51030)

18 exp Fatty Acids, Omega-3/ or omega-3*.mp. (28292)

19 exp Fish Oils/ or fish oil*.mp. (28692)

20 exp Linolenic Acids/ or linolen*.mp. (11929)

21 n3 fatty acid*.mp. (82)

22 n-3 fatty acid*.mp. (4968)

23 n3 PUFA*.mp. (79)

24 n-3 PUFA*.mp. (4127)

25 n3 polyunsaturated*.mp. (76)

26 n-3 polyunsaturated*.mp. (3894)

27 exp Eicosapentaenoic Acid/ or eicosapent?enoic*.mp. (10938)

28 docosapent?enoic*.mp. (1213)

29 exp Docosahexaenoic Acids/ or docosahex?enoic*.mp. (14520)

30 hexadecatrienoic*.mp. (78)

31 stearidonic*.mp. (251)

32 eicosatrienoic*.mp. (2218)

33 icosatrienoic*.mp. (27)

34 exp alpha-Linolenic Acid/ or alpha-linolen*.mp. (5568)

35 a-linolen*.mp. [mp=title, abstract, original title, name of substance word, subject heading word, keyword heading word, protocol supplementary concept word, rare disease supplementary concept word, unique identifier] (77)

36 icosapent?enoic*.mp. [mp=title, abstract, original title, name of substance word, subject heading word, keyword heading word, protocol supplementary concept word, rare disease supplementary concept word, unique identifier] (35)

37 eicosatetr?enoic*.mp. (4545)

38 icosatetr?enoic*.mp. [mp=title, abstract, original title, name of substance word, subject heading word, keyword heading word, protocol supplementary concept word, rare disease supplementary concept word, unique identifier] (68)

39 heneicosapent?enoic*.mp. [mp=title, abstract, original title, name of substance word, subject heading word, keyword heading word, protocol supplementary concept word, rare disease supplementary concept word, unique identifier] (5)

40 tetracosapent?enoic*.mp. [mp=title, abstract, original title, name of substance word, subject heading word, keyword heading word, protocol supplementary concept word, rare disease supplementary concept word, unique identifier] (20)

41 tetracosahex?enoic*.mp. [mp=title, abstract, original title, name of substance word, subject heading word, keyword heading word, protocol supplementary concept word, rare disease supplementary concept word, unique identifier] (30)

42 18 or 19 or 20 or 21 or 22 or 23 or 24 or 25 or 26 or 27 or 28 or 29 or 30 or 31 or 32 or 33 or 34 or 35 or 36 or 37 or 38 or 39 or 40 or 41 (52490)

43 3 and 17 and 42 (185)

44 limit 43 to randomized controlled trial (38)

**Table 2.** Search Strategy for EMBASE

1 exp sepsis/ or sepsis.mp. (246282)

2 exp septic shock/ or septic*.mp. (110615)

3 1 or 2 (273965)

Annotation: sepsis

4 enteral nutrition.mp. or exp enteric feeding/ (29899)

5 enteral feed*.mp. (7798)

6 enteric feed*.mp. (27697)

7 exp tube feeding/ or feeding tube*.mp. (4648)

8 tube feed*.mp. (6311)

9 4 or 5 or 6 or 7 or 8 (36969)

Annotation: EN

10 parenteral nutrition.mp. or exp parenteral nutrition/ (48163)

11 parenteral feed*.mp. (1774)

12 exp intravenous feeding/ or intravenous feed*.mp. (1492)

13 iv feed*.mp. (80)

14 total parenteral nutrition.mp. or exp total parenteral nutrition/ (17133)

15 total nutrient admixture*.mp. (131)

16 10 or 11 or 12 or 13 or 14 or 15 (48624)

Annotation: PN

17 exp omega 3 fatty acid/ or omega-3*.mp. (30458)

18 exp fish oil/ or fish oil*.mp. (17588)

19 exp linolenic acid/ or linolen*.mp. (14922)

20 n3 fatty acid*.mp. (122)

21 n-3 fatty acid*.mp. (5431)

22 n3 PUFA*.mp. (137)

23 n-3 PUFA*.mp. (4618)

24 n3 polyunsaturated*.mp. (89)

25 n-3 polyunsaturated*.mp. (4318)

26 exp icosapentaenoic acid/ or eicosapent?enoic*.mp. (16058)

27 icosapent?enoic*.mp. [mp=title, abstract, heading word, drug trade name, original title, device manufacturer, drug manufacturer, device trade name, keyword, floating subheading] (14501)

28 docosapent?enoic*.mp. (1721)

29 exp docosahexaenoic acid/ or docosahex?enoic*.mp. (19541)

30 hexadecatrienoic*.mp. (78)

31 exp stearidonic acid/ or stearidonic*.mp. (356)

32 exp icosatrienoic acid/ or eicosatrienoic*.mp. (1069)

33 icosatrienoic*.mp. (1250)

34 alpha-linolen*.mp. (4544)

35 a-linolen*.mp. [mp=title, abstract, heading word, drug trade name, original title, device manufacturer, drug manufacturer, device trade name, keyword, floating subheading] (261)

36 eicosatetr?enoic*.mp. (1620)

37 icosatetr?enoic*.mp. [mp=title, abstract, heading word, drug trade name, original title, device manufacturer, drug manufacturer, device trade name, keyword, floating subheading] (1068)

38 heneicosapent?enoic*.mp. [mp=title, abstract, heading word, drug trade name, original title, device manufacturer, drug manufacturer, device trade name, keyword, floating subheading] (7)

39 tetracosapent?enoic*.mp. [mp=title, abstract, heading word, drug trade name, original title, device manufacturer, drug manufacturer, device trade name, keyword, floating subheading] (19)

40 tetracosahex?enoic*.mp. [mp=title, abstract, heading word, drug trade name, original title, device manufacturer, drug manufacturer, device trade name, keyword, floating subheading] (30)

41 17 or 18 or 19 or 20 or 21 or 22 or 23 or 24 or 25 or 26 or 27 or 28 or 29 or 30 or 31 or 32 or 33 or 34 or 35 or 36 or 37 or 38 or 39 or 40 (66011)

Annotation: omega 3

42 9 or 16 (75728)

Annotation: EN/PN

43 3 and 41 and 42 (404)

44 limit 43 to randomized controlled trial (63)

**Table 3**. Search Strategy for Cochrane Library

1 MeSH descriptor: [Sepsis] explode all trees (3457)

2 MeSH descriptor: [Shock, Septic] explode all trees (519)

3 sepsis or septic* (9379)

4 #1 or #2 or #3 (10649)

5 MeSH descriptor: [Enteral Nutrition] explode all trees (1756)

6 "enteral nutrition" or "enteric nutrition" or "enteral feed*" or "enteric feed*" or "feeding tube*" or "tube feed*" (4962)

7 #5 or #6 (4962)

8 MeSH descriptor: [Parenteral Nutrition] explode all trees (1655)

9 MeSH descriptor: [Parenteral Nutrition, Total] explode all trees (792)

10 "parenteral nutrition" or "parenteral feed*" or "intravenous feed*" or "IV feed*" or "total parenteral nutrition" or "total nutrient admixture*" (3449)

11 #8 or #9 or #10 (3449)

12 MeSH descriptor: [Fatty Acids, Omega-3] explode all trees (2479)

13 MeSH descriptor: [Fish Oils] explode all trees (2799)

14 MeSH descriptor: [Linolenic Acids] explode all trees (360)

15 MeSH descriptor: [Eicosapentaenoic Acid] explode all trees (811)

16 MeSH descriptor: [Docosahexaenoic Acids] explode all trees (880)

17 MeSH descriptor: [alpha-Linolenic Acid] explode all trees (194)

18 "omega 3" or "omega-3" or "fish oil*" or "linolen*" or "n3 fatty acid*" or "n-3 fatty acid*" or "n3 PUFA*" or "n-3 PUFA*" or "n3 polyunsaturated*" or "n-3 polyunsaturated*" (5179)

19 eicosapent*enoic* or icosapent*enoic* or docosapent*enoic* or docosahex*noic* or hexadecatrienoic* or stearidonic* or eicosatrienoic* or icosatrienoic* or alpha-linolen* or a-linolen* or eicosatetr*enoic* or icosatetr*enoic* or heneicosapent*enoic* or henicosapent*enoic* tetracosapent*enoic* or tetracosahex*enoic* (2788)

20 #12 or #13 or #14 or #15 or #16 or #17 or #18 or #19 (5916)

21 #7 or #11 (7105)

22 #4 and #21 and #20 in Trials (60)

**Table 4**. Contents of Brand-name Parenteral Formulations

|  | **PARENTERAL FORMULAE** | | | |
| --- | --- | --- | --- | --- |
|  | **INTERVENTION** | | | **CONTROL** |
| Formula | Lipoplus + Nutriflex Special | Omegaven | Clinoleic | Nutriflex LipidSpecial |
| Study | Barbosa 2010 | Burkhart 2014, Grecu 2003, Gultekin 2014, Hall 2015, Khor 2011, Zhao 2011 | Gultekin 2014 | Barbosa 2010 |
| Manufacturer | B Braun | Fresenius Kabi | Baxter | B Braun |
| Energy [kcal/mL] | N/A | 1.12 | 2 | N/A |
| Osmolality [mOsm/L] | N/A | 308 - 376 | 345 | N/A |
| Proteins [g/L] | 56 | 0 | 0 | 57.4 |
| Carbohydrates [g/L] | 195 | 0 | 0 | 144 |
| Lipids [g/L] | 40 | 100 | 200 | 40 |
| Omega-3 [g/L] | N/A | N/A | 2.3 | N/A |
| EPA [g/L] | 2.5% of FA | 12.5 - 28.2 | ~0.04 | 0 |
| DHA [g/L] | 1.1% of FA | 14.4 - 30.9 | 2.3 | 0 |
| EPA + DHA [g/L] | 3.6% of FA | 26.9 - 59.1 | 2.34 | 0 |
| GLA [g/L] | N/A | N/A | ~0.2 | N/A |
| n6:n3 ratio | N/A | N/A | 8-9:1 | N/A |
| Vitamins and minerals (amount/L) | N/A | N/A | N/A | N/A |

Nutritional data were extracted from individual studies where available, and otherwise supplemented with manufacturer data. *EPA* eicosapentaenoic acid, *DHA* docosahexaenoic acid*, GLA* gamma-linoleic acid*, n6* omega-6*, n3* omega-3, *N/A* not available.

**Table 5**. Contents of Brand-name Enteral Formulations

|  | **ENTERAL FORMULAE** | | | | | | | |
| --- | --- | --- | --- | --- | --- | --- | --- | --- |
|  | **INTERVENTION** | | | **CONTROL** | | | | |
| Formula | Impact | Oxepa | Oxepa | Osmolite HN | Ensure Plus HN | Ensure Plus HN | Ensure Liquid | Precipitene Hiperproteico |
| Study | Bower 1995, Galban 2000 | Pontes-Arruda 2011, Shirai 2015 | Grau-Carmona 2011 | Bower 1995 | Pontes-Arruda 2011 | Grau-Carmona 2011 | Shirai 2015 | Galban 2000 |
| Manufacturer | Sandoz Nutrition, Minneapolis; Novartis Nutrition, Spain | Abbott Laboratories, Chicago IL; Abbott Nutrition, Tokyo Japan | Abbott Laboratories, Madrid Spain | Sandoz Nutrition, Minneapolis | Abbott Laboratories, Chicago IL | Abbott Labs, Madrid Spain | Abbott Nutrition, Tokyo Japan | Novartis Nutrition, Switzerland |
| Energy [kcal/mL] | 1.0 | 1.5 | 1.52 | 1.0 | 1.5 | 1.0 | 1.0 | 1.2 |
| Osmolality [mOsm/L] | 375 | 384 | 385 | 375 | 392 | 319 | 330 | N/A |
| Proteins [g/L] | 56 | 62.5 | 52.5 | 44 | 62.7 | 66.6 | 35.2 | 66.2 |
| Carbohydrates [g/L] | 132 | 106 | 27.94% | 141 | 204 | 53.98% | 137.2 | 148.2 |
| Lipids [g/L] | 28 | 93.7 | 55.58% | 37 | 49.1 | 30.10% | 35.2 | 40.2 |
| Omega-3 [g/L] | 2.1 | 10 | 12.2 | 0 | 1.5 | 1.3 | 0 | 0 |
| EPA [g/L] | N/A | 4.5-5.1 | N/A | 0 | 0 | 0 | 0 | 0 |
| DHA [g/L] | N/A | 2.0-2.2 | 2.4 | 0 | 0 | 0 | 0 | 0 |
| EPA + DHA [g/L] | 2.6 | 6.5-7.3 | N/A | 0 | 0 | 0 | 0 | 0 |
| GLA [g/L] | 0 | 4.1-4.3 | 0.1 | 0 | 0 | 0 | 0 | 0 |
| n6:n3 ratio | 1.4:1 | 1.6-1.85 : 1 | 1.5:1 | N/A | 3.8:1 | 5.8:1 | 44:1 | N/A |
| Vitamins and minerals (amount/L) | L-Arginine 12.5g, Vitamin A (Retinol 1000mcg, Beta-Carotene 2000mcg), Vitamin C 80mg, Calcium 800mg, Iron 12mg, Vitamin D 13.2 mcg, Vitamin E 40mg, Vitamin K 68mcg, Thiamine 2mg, Riboflavin 1.7mg, Niacin 20mg, Vitamin B6 1.6mg, Folic Acid 400mcg, Vitamin B12 8mg, Biotin 200mcg, Pantothenic Acid 6.8mg, Phosphorus 800mg, Iodine 100mcg, Magnesium 272mg, Zinc 15.2mg, Selenium 100mcg, Copper 1.7 mg, Manganese 2mg, Chromium 100mg, Molybdenum 200mcg , Chloride 1300mg, Choline 272mg, Water 852mL | Vitamin A 11910 IU, Beta-Carotene 5mg, Vitamin D 425IU, Vitamin E 320 IU, Vitamin K 85mcg, Vitamin C 850mg, Folic Acid 850mcg, Vitamin B1 3.2mg, Riboflavin 3.6 mg, Vitamin B6 4.3mg, Vitamin B12 13mcg, Niacin 43mg, Choline 635mg, Biotin 635mcg, Pantothenic Acid 22mg, Sodium 1310mg, Potassium 1960 mg, Chloride 1690 mg, Calcium 1060mg, Phosphorus 1060mg, Magnesium, 425mg, Iodine 160 mcg, Manganese 5.3 mg, Copper 2.2mg, Zinc 24mg, Iron 20mg, Selenium 74mcg, Chromium 130mcg, Molybdenum 160mcg | Vitamin D3 425IU, Vitamin E 317IU, Vitamin K1 101mcg, Vitamin C 844mcg, Zinc 18mg, Iodine 160 mcg, Selenium 77 mcg, Chromium 130 mcg, Molybdenum 160mcg | Vitamin A 3572 IU, Vitamin E 32 IU, Vitamin C 215 mg, Folate 429 ug, Vitamin B6 2.13 mg, Vitamin B12 6.4 ug, Zinc 16mg, Selenium 50 ug | Vitamin E 39IU, Vitamin C 130mg, B-Carotene 345mg, Taurine 150mg, L-Carnitine 120mg, Vitamin A 4167IU, Vitamin D 400IU, Vitamin K 80ug, Folic Acid 400ug, Thiamine 2.8mg, Riboflavin 3.4mg, B6 3.9mg, B12 4.0mg, Niacin 29mg, Choline 800mg, Biotin 72ug, Pantothenic Acid 13mg, Sodium 1400mg, Potassium 1650mg, Chloride 1450mg, Calcium 1000mg, Phosphorous 1000mg, Magnesium 310mg, Iodine 150mg, Mn 5mg, Copper 2.5mg, Zinc 19mg, Iron 22mg, Selenium 70ug, Chromium 100, Mo 150ug | Vitamin D3 290IU, Vitamin E 32IU, Vitamin K1 52mcg, Vitamin C 100mcg, Zinc 12mg | Palmitate 2500 IU, Vitamin D3 200 IU, Vitamin E 45 mg, Vitamin C 152 mg, Folic Acid 200ug, Vitamin B1 1.5mg, Vitamin B2 1.7 mg, Vitamin B6 2mg, Vitamin B12 6ug, Niacin 20mg, Pantothenic Acid 5mg, Biotin 15.2ug, Sodium 800mg, Potassium 1480mg, Chloride 1360mg, Calcium 520mg, Phosphorus 520mg, Magnesium 200mg, Iron 9mg, Zinc 15mg, Copper 1000ug | Sodium 600mg, Chloride 1150mg, Potassium 996mg, Calcium 750mg, Phosphorus 750mg, Magnesium 250mg, Iron 9mg, Iodine 75ug, Zinc 9mg, Flouride 0.76mg, Copper 1mg, Manganese 1.2mg, Selenium 35.4ug, Chromium 54.6ug, Molybdenum 84.6ug, Vitamin A 0.6mg, Vitamin D 2.4ug, Vitamin E 6mg, Vitamin K 40ug, Thiamin 0.8mg, Riboflavin 0.8mg, Niacin 9.4mg, Vitamin B6 1mg, Folic Acid 200ug, Vitamin B12 1.4ug, Biotin 75ug, Pantothenic Acid 4mg, Vitamin C 40mg, Choline 100mg |

Nutritional data were extracted from individual studies where available, and otherwise supplemented with manufacturer data. *EPA* eicosapentaenoic acid, *DHA* docosahexaenoic acid*, GLA* gamma-linoleic acid*, n6* omega-6*, n3* omega-3, *N/A* not available.

**Figure 1.** Subgroup Analysis for Mortality Outcome


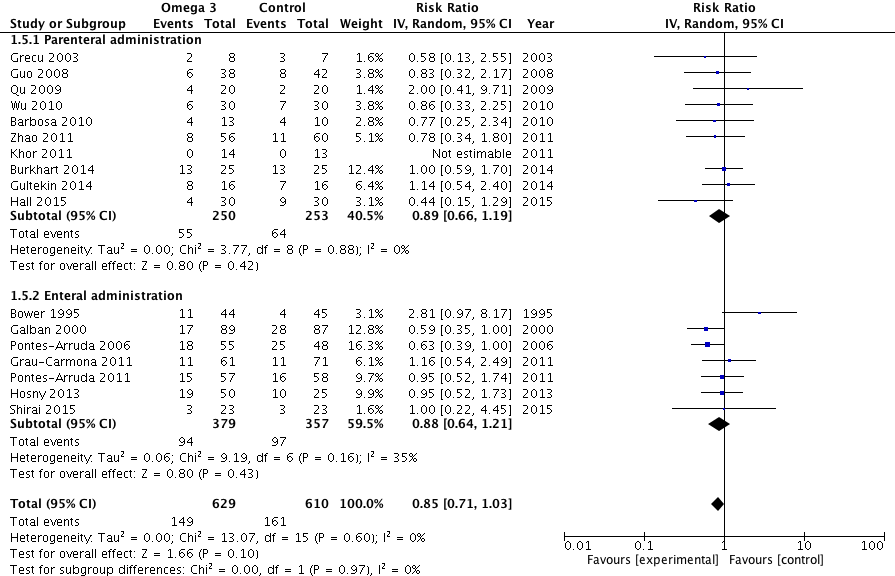


Comparison of studies using parenteral versus enteral administration of omega-3 demonstrated no significant subgroup differences. *IV* inverse variance.

**Table 5**. Sensitivity Analyses for Mortality Outcome

| **Mortality** | Omega-3: events / total | Control:  events / total | RR [95% CI] | Overall effect: *P* | Heterogeneity: *P, I²* |
| --- | --- | --- | --- | --- | --- |
| No exclusions  (17 studies) | 149 / 629 | 161 / 610 | RR: 0.85 [0.71, 1.03]  + FE: 0.85 [0.71, 1.03]  OR: 0.80 [0.61, 1.05] | 0.10  0.10  0.11 | 0.60, 0%  0.60, 0%  0.54, 0% |
| Excluding trials that did not clearly specify ICU admission under eligibility criteria (16 studies) | 145 / 609 | 159 / 590 | RR: 0.84 [0.70, 1.02] | 0.08 | 0.61, 0% |
| Excluding trials published in abstract form (16 studies) | 147 / 621 | 158 / 603 | RR: 0.86 [0.71, 1.04] | 0.11 | 0.54, 0% |
| Excluding trials that used control formulae containing omega-3 (15 studies) | 123/511 | 134/481 | RR: 0.82 [0.67, 1.01] | 0.06 | 0.51, 0% |
| Excluding trials that did not administer the control group a placebo (13 studies) | 105/468 | 118/470 | RR: 0.85 [0.68, 1.06] | 0.15 | 0.43, 1% |
| Excluding trials that used per-protocol analysis (7 studies) | 53 / 212 | 51 / 190 | RR: 0.89 [0.65, 1.22] | 0.46 | 0.77, 0% |
| Excluding trials that were not explicitly blinded to patients and healthcare personnel (6 studies) | 54 / 194 | 55 / 187 | RR: 0.96 [0.61, 1.53] | 0.88 | 0.12, 46% |

Exclusion of trials using per-protocol analysis revealed similar results to the primary analysis. *CI* confidence interval, *FE* fixed-effects, *ICU* intensive care unit, *OR* odds ratio, *RR* relative risk.

**Table 6**. Sensitivity Analyses for ICU Length of Stay Outcome

| **ICU Length of Stay** | Omega-3: total | Control: total | MD [95% CI] | Overall effect: *P* | Heterogeneity: *P, I²* |
| --- | --- | --- | --- | --- | --- |
| No exclusions  (12 studies) | 469 | 456 | MD: -3.79 [-5.49, -2.09]  + FE: -3.80 [-4.35, -3.25] | <0.0001  <0.00001 | <0.00001, 82%  <0.00001, 82% |
| Excluding trials published in abstract form (11 studies) | 461 | 449 | MD: -3.54 [-5.37, -1.72] | 0.0001 | <0.00001, 83% |
| Excluding trials that used control formulae containing omega-3 (10 studies) | 351 | 327 | MD: -3.65 [-5.59, -1.71] | 0.0002 | <0.00001, 84% |
| Excluding trials that did not administer the control group a placebo (9 studies) | 333 | 341 | MD: -4.22 [-6.44, -1.99] | 0.0002 | <0.00001, 79% |
| Excluding trials that did not report SD (7 studies) | 301 | 281 | MD: -3.91 [-5.97, -1.84] | 0.0002 | <0.00001, 87% |
| Excluding trials that used per-protocol analysis (5 studies) | 167 | 145 | MD: -3.96 [-5.94, -1.99] | <0.0001 | 0.010, 70% |
| Excluding trials that were not explicitly blinded to patients and healthcare personnel (3 studies) | 79 | 78 | MD: -3.63 [-7.85, 0.60] | 0.09 | 0.001, 85% |

Exclusion of trials using per-protocol analysis revealed similar results to the primary analysis. Exclusion of trials that did not explicitly blind patients and healthcare personnel to the intervention produced a non-significant effect. *CI* confidence interval, *FE* fixed-effects, *ICU* intensive care unit, *LOS* length of stay, *MD* mean difference, *SD* standard deviation.

**Table 7**. Sensitivity Analyses for Duration of Mechanical Ventilation Outcome

| **Duration of Mechanical Ventilation** | Omega-3: total | Control: total | MD [95% CI] | Overall effect: *P* | Heterogeneity: *P, I²* |
| --- | --- | --- | --- | --- | --- |
| No exclusions (7 studies) | 254 | 241 | MD: -2.27 [-4.27, -0.27]  With FE: -2.01 [-3.17, -0.84] | 0.03  0.0007 | 0.02, 60%  0.02, 60% |
| Excluding trials published in abstract form (6 studies) | 246 | 234 | MD: -2.32 [-4.86, 0.22] | 0.07 | 0.01, 67% |
| Excluding trials that did not administer the control group a placebo feed (6 studies) | 204 | 216 | MD: -2.10 [-4.48, 0.29] | 0.08 | 0.01, 65% |
| Excluding trials that used control formulae containing omega-3 (5 studies) | 183 | 152 | MD: -2.47 [-3.98, -0.97] | 0.001 | 0.31, 16% |
| Excluding trials that did not stratify randomization by mechanical ventilation (4 studies) | 105 | 111 | MD: -1.78 [-4.39, 0.83] | 0.18 | 0.05, 61% |
| Excluding trials that used per-protocol analysis (3 studies) | 81 | 55 | MD: -3.18 [-4.70, -1.67] | <0.0001 | 0.63, 0% |
| Excluding trials that did not report SD (3 studies) | 147 | 119 | MD: -1.97 [-3.86, -0.07] | 0.04 | 0.23, 32% |
| Excluding trials that were not explicitly blinded to patients and healthcare personnel (2 studies) | 18 | 25 | MD: -4.63 [-10.00, 0.75] | 0.09 | 0.07, 70% |

Exclusion of trials using per-protocol analysis and trials that did not report standard deviations (necessitating conversion from standard error, confidence interval, and interquartile ranges) revealed similar results to the primary analysis. Exclusion of trials published in abstract form, trials that did not stratify randomization by mechanical ventilation, and trials that were not explicitly blinded to patients and healthcare personnel produced a non-significant effect. *CI* confidence interval, *DMV* duration of mechanical ventilation, *FE* fixed-effects, *MD* mean difference, *SD* standard deviation.

**Figure 2.** Funnel Plot for Mortality Outcome


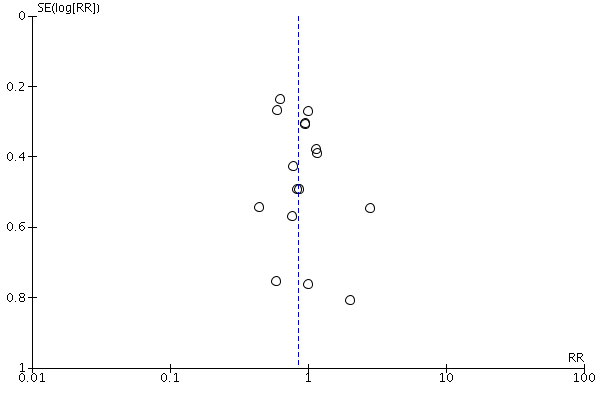


Visual inspection did not reveal small-study effects.

**Figure 3**. Funnel Plot for ICU Length of Stay Outcome


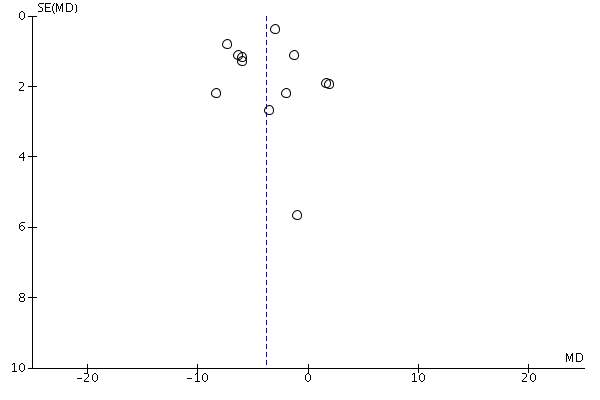


Visual inspection did not reveal small-study effects.

**Table 8.** PRISMA Checklist

| **TITLE** | | |  |
| --- | --- | --- | --- |
| Title | 1 | Identify the report as a systematic review, meta-analysis, or both. | 1 |
| **ABSTRACT** | | |  |
| Structured summary | 2 | Provide a structured summary including, as applicable: background; objectives; data sources; study eligibility criteria, participants, and interventions; study appraisal and synthesis methods; results; limitations; conclusions and implications of key findings; systematic review registration number. | 3  Systematic review registration number N/A |
| **INTRODUCTION** | | |  |
| Rationale | 3 | Describe the rationale for the review in the context of what is already known. | 5 |
| Objectives | 4 | Provide an explicit statement of questions being addressed with reference to participants, interventions, comparisons, outcomes, and study design (PICOS). | 5 |
| **METHODS** | | |  |
| Protocol and registration | 5 | Indicate if a review protocol exists, if and where it can be accessed (e.g., Web address), and, if available, provide registration information including registration number. | 5 |
| Eligibility criteria | 6 | Specify study characteristics (e.g., PICOS, length of follow-up) and report characteristics (e.g., years considered, language, publication status) used as criteria for eligibility, giving rationale. | 5 |
| Information sources | 7 | Describe all information sources (e.g., databases with dates of coverage, contact with study authors to identify additional studies) in the search and date last searched. | 5 |
| Search | 8 | Present full electronic search strategy for at least one database, including any limits used, such that it could be repeated. | Additional File 1: Tables S1-S3 |
| Study selection | 9 | State the process for selecting studies (i.e., screening, eligibility, included in systematic review, and, if applicable, included in the meta-analysis). | 5-6 |
| Data collection process | 10 | Describe method of data extraction from reports (e.g., piloted forms, independently, in duplicate) and any processes for obtaining and confirming data from investigators. | 6 |
| Data items | 11 | List and define all variables for which data were sought (e.g., PICOS, funding sources) and any assumptions and simplifications made. | 5-7 |
| Risk of bias in individual studies | 12 | Describe methods used for assessing risk of bias of individual studies (including specification of whether this was done at the study or outcome level), and how this information is to be used in any data synthesis. | 6 |
| Summary measures | 13 | State the principal summary measures (e.g., risk ratio, difference in means). | 7 |
| Synthesis of results | 14 | Describe the methods of handling data and combining results of studies, if done, including measures of consistency (e.g., I^2^) for each meta-analysis. | 7 |
| Risk of bias across studies | 15 | Specify any assessment of risk of bias that may affect the cumulative evidence (e.g., publication bias, selective reporting within studies). | 7 |
| Additional analyses | 16 | Describe methods of additional analyses (e.g., sensitivity or subgroup analyses, meta-regression), if done, indicating which were pre-specified. | 7-8 |
| **RESULTS** | | | |
| Study selection | 17 | Give numbers of studies screened, assessed for eligibility, and included in the review, with reasons for exclusions at each stage, ideally with a flow diagram. | 8-9, Figure 1 |
| Study characteristics | 18 | For each study, present characteristics for which data were extracted (e.g., study size, PICOS, follow-up period) and provide the citations. | 9-10, Table 1 |
| Risk of bias within studies | 19 | Present data on risk of bias of each study and, if available, any outcome level assessment (see item 12). | 15, Figure 2 |
| Results of individual studies | 20 | For all outcomes considered (benefits or harms), present, for each study: (a) simple summary data for each intervention group (b) effect estimates and confidence intervals, ideally with a forest plot. | Figures 3-5 |
| Synthesis of results | 21 | Present results of each meta-analysis done, including confidence intervals and measures of consistency. | 17, Table 2, Figures 3-5 |
| Risk of bias across studies | 22 | Present results of any assessment of risk of bias across studies (see Item 15). | 19, Additional File 1: Figures S2-S3 |
| Additional analysis | 23 | Give results of additional analyses, if done (e.g., sensitivity or subgroup analyses, meta-regression [see Item 16]). | 17-19, Additional File 1: Figure S1 and Tables S5-S7 |
| **DISCUSSION** | | | |
| Summary of evidence | 24 | Summarize the main findings including the strength of evidence for each main outcome; consider their relevance to key groups (e.g., healthcare providers, users, and policy makers). | 19 |
| Limitations | 25 | Discuss limitations at study and outcome level (e.g., risk of bias), and at review-level (e.g., incomplete retrieval of identified research, reporting bias). | 21 |
| Conclusions | 26 | Provide a general interpretation of the results in the context of other evidence, and implications for future research. | 22 |
| **FUNDING** | | | |
| Funding | 27 | Describe sources of funding for the systematic review and other support (e.g., supply of data); role of funders for the systematic review. | 23 |

*From:*  Moher D, Liberati A, Tetzlaff J, Altman DG, The PRISMA Group (2009). Preferred Reporting Items for Systematic Reviews and Meta-Analyses: The PRISMA Statement. PLoS Med 6(7): e1000097. doi:10.1371/journal.pmed1000097
